# Event and Comment

**Published:** 1913-02-15

**Authors:** 


### DENTAL REGISTER.

**Vol. LXVII. February 15, 1913. No. 2**

### EVENT AND COMMENT.

PYORRHEA AND ITS TREATMENT. We are printing in this issue an article on this much discussed subject from the pen of Professor Thomas B. Hartzell, of the University of Minnesota, which to our notion, sets forth the etiology and treatment of this disease in the most scientific and up-to-date form that we have ever seen. The article is necessarily long, but it will well repay not only a casual reading, but if carefully studied it will give any one an intelligent conception of treatment that will insure success and convince the skeptical that pyorrhea is a curable disease. The multiplicity of "cures" for this disease which ignore its etiology and claim specific action without surgical interference have come short of our expectations, and the disappointed have concluded that there is no cure. This article may not definitely name the *cure* and may also disappoint, but it will certainly indicate a rational treatment based on a knowledge of conditions which any intelligent and skillful operator can apply as a most successful remedy. The disease can be eradicated on the basis here outlined and conditions made that will with the help of natural repair processes, predispose to as complete a cure as the age of the patient and extent of the injury can be hoped for in similar affections. The treatment involves persistent and skillful operative proceedure, and any one who is expecting a treatment for this disease that is easy of application will be disappointed in this article, as he is certain to be with all automatic remedies.

DENTAL LIBRARIES. In a recent issue of the Bulletin of the Illinois State Dental Society we notice a report of the condition of the dental library in the John Crearar Library of Chicago. From this report we notice that a complete file of eleven of our dental periodicals has been collected. This is gratifying as this form of early literature has almost disappeared and it is not likely that many more complete files of our early journals can be got together. The Chicago dentists ought to see to it that this library is made as complete as it is possible to make it. There will come a time when this literature will be of much interest and value. So much of our literature has been contributed only through the dental journals that it will be necessary to preserve all our periodic literature as carefully as possible. A number of our colleges have fairly complete libraries; but the effort at Chicago, like that of Columbus, Ohio, to accumulate our professional literature should be encouraged. Every important center ought at least to have a library of present day dental publications for the use of those who wish to prepare papers for dental societies, where they can get a resume of present day literature.

REORGANIZATION OF THE NATIONAL DENTAL ASSOCIATION. Seventeen of the twenty-seven local and district societies of Illinois have agreed to unite with the new National body. and it is confidently expected the other ten will do the same when they have had their annual meetings. The news from other States is likewise encouraging. If Illinois gets all of her 1700 members and Ohio her 900 members, the present membership of the National will be exceeded by these two States.

